# Diverse mechanisms underlying the fetal growth course in gastroschisis and omphalocele

**DOI:** 10.1016/j.xagr.2023.100238

**Published:** 2023-06-04

**Authors:** Sofia Amylidi-Mohr, Melanie Wyss, Daniel Surbek, Luigi Raio, Beatrice Mosimann

**Affiliations:** 1 Department of Obstetrics, University Hospital of Bern, University of Bern, Bern, Switzerland (Dr Amylidi-Mohr, Ms Wyss, Messrs Surbek and Raio, and Ms Mosimann); 2 University Hospital of Basel, University of Basel, Basel, Switzerland (Dr Amylidi-Mohr and Ms Mosimann)

**Keywords:** abdominal wall defects, birthweight, growth restriction, placental weight, omphalocele and gastroschisis

## Abstract

**BACKGROUND:**

Gastroschisis and omphalocele are the 2 most common congenital fetal abdominal wall defects. Both malformations are commonly associated with small-for-gestational-age neonates. However, the extent and causes of growth restriction remain controversial in both gastroschisis and omphalocele without associated malformations or aneuploidy.

**OBJECTIVE:**

This study aimed to examine the role of the placenta and the birthweight–to–placental weight ratio in fetuses with abdominal wall defects.

**STUDY DESIGN:**

This study included all cases of abdominal wall defects examined at our hospital between January 2001 and December 2020, retrieving the data from the hospital's software. Fetuses with any other combined congenital anomalies, known chromosomal abnormalities, or lost to follow-up were excluded. Overall, 28 singleton pregnancies with gastroschisis and 24 singleton pregnancies with omphalocele met the inclusion criteria. Patient characteristics and pregnancy outcomes were reviewed. The primary outcome was to investigate the association between birthweight and placental weight in pregnancies with abdominal wall defects as measured after delivery. To correct for gestational age and to compare total placental weights, ratios between the observed and expected birthweights for the given gestational age in singletons were calculated. The scaling exponent β was compared with the reference value of 0.75. Statistical analysis was performed using GraphPad Prism (version 8.2.1; GraphPad Software, San Diego, CA) and IBM SPSS Statistics. A *P* value of <.05 indicated statistical significance.

**RESULTS:**

Women pregnant with a fetus with gastroschisis were significantly younger and more often nulliparous. In addition, in this group, the gestational age of delivery was significantly earlier and almost exclusively for cesarean delivery. Of 28 children, 13 (46.7%) were born small for gestational age, only 3 of them (10.7%) had a placental weight <10th percentile. There is no correlation between birthweight percentiles and placental weight percentiles (*P*=not significant). However, in the omphalocele group, 4 of 24 children (16.7%) were born small for gestational age (<10th percentile), and all children also had a placental weight <10th percentile. There is a significant correlation between birthweight percentiles and placental weight percentiles (*P*<.0001). The birthweight–to–placental weight ratio differs significantly between pregnancies diagnosed with gastroschisis and pregnancies diagnosed with omphalocele (4.48 [3.79–4.91] vs 6.05 [5.38–6.47], respectively; *P*<.0001). Allometric metabolic scaling revealed that placentas complicated by gastroschisis and placentas complicated by omphalocele do not scale with birthweight.

**CONCLUSION:**

Fetuses with gastroschisis displayed impaired intrauterine growth, which seemed to differ from the classical placental insufficiency growth restriction.

**AJOG Global Reports at a Glance**

**Why was this study conducted?**

This study aimed to better understand the pathophysiology of growth restriction and placenta dysfunction in abdominal wall defects (AWDs).

**Key findings**

The underlying mechanism of growth restriction seems to be different for the 2 common forms of AWDs. In small newborns with omphalocele, growth restriction is associated with small placental size, suggestive of placental malfunction. In children with gastroschisis, the underlying mechanism remains less clear.

**What does this add to what is known?**

Depending on the nature of the AWD, obstetricians may adapt the course of management. The altered metabolic scaling is a marker for a higher percentage of adverse outcomes, and these pregnancies require closer monitoring, independent of the fetal malformations.

## Introduction

Gastroschisis and omphalocele are the 2 most common congenital fetal abdominal wall defects (AWDs), with a prevalence of 3 in 10,000 births.^1^ Although gastroschisis is characterized by a full-thickness paraumbilical defect in the abdominal wall with herniation of viscera, usually bowel loops, without a covering membrane or sac, omphalocele is a defect with intra-abdominal contents present, which herniate within a peritoneal sac into the amniotic cavity through the base of the umbilical cord.^1^ Because of the dissimilar pathophysiological mechanism, we expect causes of fetal growth restriction (FGR) to differ in these 2 identities.

FGR because of placental insufficiency arises from compromise of the uterine circulation of the placenta,^2^ leading to chronic fetal hypoxia and hypoglycemia.^3^ Placentas complicated by FGR are smaller throughout gestation and display maldevelopment of the placental villi and the fetal vasculature within these villi.^4^ In addition, birthweight (BW)–to–placental weight (PW) ratio, defined as gram neonate per gram placenta, reflects a marker of placental efficiency. An elevated neonatal to placental weight ratio (n-to-p ratio) could be a marker for increased nutrient transfer to the fetus, which, despite its normal weight, seems to be at risk by outgrowing its placenta, whereas the implications of low n-to-p ratio are less understood.^5^^,^^6^ A similar approach to assess placental efficacy is to calculate the metabolic scaling exponent ß, which reflects the fractal structure of the placental vasculature. The model of metabolic scaling reveals a possible mechanism on how the placenta translates into neonatal mass, thus metabolism into organism.^7^^,^^8^ Understanding the pathophysiology of growth restriction in children with AWD remains important. It could potentially influence the management. To date, fetuses with FGR are often delivered prematurely; however, in children with gastroschisis, this is associated with increased gastrointestinal morbidity.^9^, ^10^, ^11^ As there are still only limited and inconsistent data available for AWDs and growth development, this study aimed to examine the association between neonatal weight and PW in fetuses with AWDs. Moreover, to estimate placental function, assessing the weight, calculating the BW-to-PW ratio, and analyzing whether the scaling exponent β in our population is close to 0.75 would be congruent with optimal placental metabolic efficiency.^7^

## Materials and Methods

This is an observational study of retrospective data on fetuses with AWDs diagnosed prenatally at the Department of Obstetrics and Gynecology of the University Hospital Bern. The study was conducted according to the Strengthening the Reporting of Observational Studies in Epidemiology guidelines.^12^

We included all cases of AWDs attended to in our hospital between January 2001 and December 2020. Fetuses with any other combined congenital anomalies, known chromosomal abnormalities, or lost to follow-up (LTFU) were excluded. The study was approved by the Ethics Committee of Bern. Data were stored in a dedicated database (ViewPoint 5; GE HealthCare GmbH, Munich, Germany) and subsequently prospectively retrieved for analysis. The diagnoses of gastroschisis and omphalocele were made after consulting a fetomaternal specialist in our department to minimize interobserver variability.

Patient characteristics were reviewed, such as median maternal age, median maternal weight, median maternal height, median maternal body mass index at 12 weeks of gestation, parity, smoking during pregnancy, conception, chronic hypertension, and preexisting diabetes mellitus. Furthermore, pregnancy outcomes, such as mode of delivery, median gestational age (GA) at delivery, median Apgar score, median arterial pH, and pregnancy complications, were recorded.

The main outcome was the neonatal weight and PW of neonates with AWDs as measured after delivery. The percentiles were calculated with a unified modeling methodology by the Fetal Medicine Foundation's fetal and neonatal population weight charts.^13^ PW was assessed in the delivery ward after the removal of fetal membranes, the umbilical cord, and blood clots. The placentas were weighed by midwives using an accurate electronic bench scale (PBA655-A6; Mettler Toledo, Greifensee, Switzerland). The PW scale and percentiles were calculated according to Thompson et al.^14^

To correct for GA and to compare total PWs, ratios between the observed and expected BWs for the given GA in singletons were calculated. Pinar et al^15^ have published PW reference values in singleton pregnancies, allowing calculation of the expected PW for a given GA: the polynomial regression equations that best fitted the mean PW (y) for singletons at a given GA (x) was derived from Pinar et al^15^ and yielded y=−531.3+33.22x−0.1623 × 2.

Analogous calculations were performed regarding the total BW. The expected mean BW (x') for a determined GA (y') was derived from a third-order polynomial regression (y'=27,789−2790x'+91.49x'2−0.9235x'3) based on published reference values for singletons by Yudkin et al.^16^ To verify the neonatal-placental scaling exponent ß, the metabolic scaling equation was applied and fitted as described by Salafia et al.^7^ Because human neonatal BW does not scale linearly with the PW but follows the rules of the allometric metabolic scaling model described by Kleiber's^17^ law and Ahern's adaptation for the fetoplacental unit we considered following the formula: PW=α (BW)β, which reveals the relationship between PW and BW under the hypothesis that the placenta and the fetus interact similar to a fractal supply system. We considered as reference the β value close to the value of 0.75, which has been previously described as normal in allometric metabolic studies in singleton pregnancies.^18^ The data were fitted by ordinary linear least-square regression using the curve-fitting tool of the statistical software. Briefly, Ahern's power function relationship, PW=a (BW)^β^, was transformed in a linear form by applying the natural logarithm to both sides: Ln (PW)=Lna+ß × Ln (BW).

Continuous data were assessed for normality using the D'Agostino-Pearson normality test. If data were distributed normally, a 2-tailed *t* test was performed, assuming both populations had similar standard deviation. Normally distributed data are presented as mean with a 95% confidence interval. If data were not distributed normally, a 2-tailed Mann-Whitney *U* test was performed to compare ranks. Data with nonnormal distribution are presented as median and interquartile range. Categorical data were analyzed using the Fisher exact test when comparing 1 or 2 variables and using the chi-square test when comparing more than 2 variables. These data are presented as number (percentage). The Spearman rank correlation and linear logistic regression were used to assess the relationship between GA, BW, and PW. Statistical analysis was performed using GraphPad Prism (version 8; GraphPad Software, San Diego, CA) and IBM SPSS Statistics (version 25; IBM Corp, Armonk, NY). A *P* value of <.05 indicated statistical significance. An approval from the local ethical committee was obtained (identification number: 2019-01828).

## Results

Between January 2001 and December 2020, we diagnosed 212 fetuses with AWDs. After excluding twin pregnancies, complex anomalies, terminations of pregnancy, intrauterine fetal demise (IUD) before viability, and LTFU, 68 cases remained. Of those cases, 31 were diagnosed with omphalocele, and 37 were diagnosed with gastroschisis. Of note, 5 pregnancies complicated by omphalocele were excluded because of aneuploidies; in 11 pregnancies, PW was not available. Overall, we included 28 singleton pregnancies with gastroschisis and 24 singleton pregnancies with omphalocele in the study.

Patient characteristics and pregnancy outcome data are depicted in Table 1. Women pregnant with a child with gastroschisis were significantly younger and more often nulliparous. In the group with gastroschisis, the GA of delivery was significantly earlier and almost exclusively for cesarean delivery. Concerning pregnancy outcomes, only the median blood loss showed a significant difference between the 2 groups. However, both gestational diabetes mellitus and preeclampsia occurred twice only in women expecting a child with omphalocele (*P*=not significant [NS]).

**Table 1:** Demographics and pregnancy outcomes of the study population grouped according to type of fetal abdominal wall defect

Variable	Gastroschisis (n=28)	Omphalocele (n=24)	*P* value
Age (y)	25.1 (20.9–28.8)	32.4 (29.3–36.1)	<.0001
Median maternal weight (kg)	61.0 (55.0–66.5)	62.0 (58.5–71.3)	NS
Median maternal height (cm)	165.0 (161.0–168.0)	164.5 (161.5–170.5)	NS
Median maternal BMI (kg/m^2^)	21.9 (19.9–24.3)	22.5 (19.9–27.3)	NS
Parity			
Nulliparous	24 (86)	12 (50)	.0074
Multiparous	4 (14)	12 (50)	.0074
Cigarette smoker	5 (18)	0 (0)	NS
Drug use disorder	3 (11)	0 (0)	NS
Conception			
Spontaneous	28 (100)	22 (91.7)	NS
ART	0 (0)	2 (8)	NS
Chronic hypertension	0 (0)	1 (4)	NS
Preexisting diabetes mellitus	0 (0)	0 (0)	NS
Mode of delivery			
Vaginal	1 (4)	5 (20.8)	NS
Operative vaginal delivery	0 (0)	0 (0)	NS
Cesarean delivery	27 (96)	19 (79.2)	NS
Median GA at delivery (wk)	36.2 (35.0–37.0)	37.9 (36.7–38.4)	.0001
Delivery before 37 wk	21 (75)	7 (29)	.0019
Median Apgar score			
1 min	8 (8–8)	8 (5–8)	NS
5 min	9.0 (8.0–9.0)	8.5 (7.3–9.0)	NS
Median arterial pH	7.33 (7.29–7.36)	7.32 (7.32–7.35)	NS
Pregnancy complications			
Preeclampsia	0 (0)	2 (8.3)	NS
Gestational diabetes mellitus	0 (0)	2 (8.3)	NS
Blood loss	500 (300–520)	500 (425–700)	NS
PPH≥1000 mL	0 (0)	3 (12.5)	NS

Data are presented as median (interquartile range) or number (percentage), unless otherwise indicated. In the comparison between the group with gastroschisis and the group with omphalocele, the Fisher exact test was used for categorical variables, and the Mann-Whitney test was used for continuous variables. A *P* value of <.05 is considered significant.

*ART*, assisted reproductive technology; *BMI*, body mass index; *GA*, gestational age; *NS*, not significant; *PPH*, postpartum hemorrhage.

**Figure 1:**
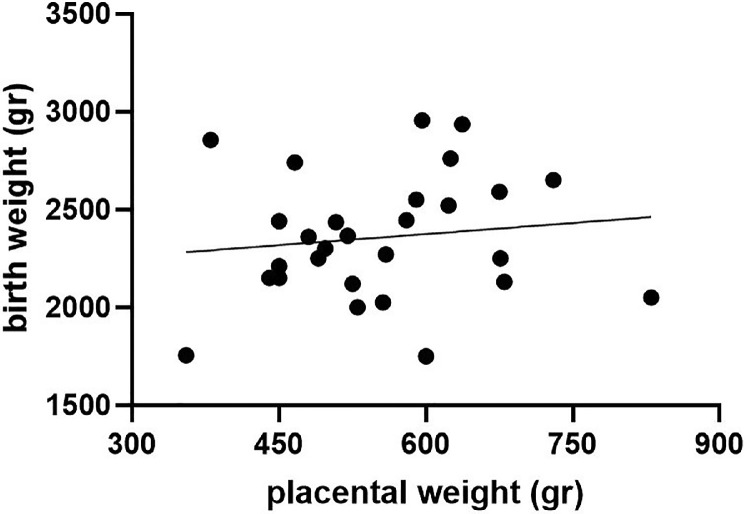
Correlation of birthweight and placental weight in children born with gastroschisis

Of the 28 children diagnosed with gastroschisis, 1 child was stillborn at 37 weeks of gestation with a BW of 2740 g (29th percentile), all other neonates were live born, and all were included in this analysis. BWs, PWs, and BW-to-PW ratios are described in Table 2. Of 28 children, 13 (46.4%) were born SGA, and only 3 of them (10.7%) had a PW <10th percentile. There is no correlation between BW percentiles and PW percentiles (*P*=NS) (Figure 1).

**Table 2:** BWs, PWs, and their percentiles and ratios grouped according to type of fetal abdominal wall defect

Variable	Gastroschisis (n=28)	Omphalocele (n=24)	*P* value
SGA according to BW	n=13	n=4	
<10th percentile	13 (46.4)	4 (16.7)	.037
<5th percentile	11 (39.3)	3 (12.5)	NS
PW<10th percentile	3 (10.7)	10 (41.7)	.022
Median BW (g)	2330 (2135–2580)	3055 (2645–3363)	.0001
Median BW percentile	11 (2.3–30.8)	44 (19.0–71.8)	.0013
Median PW	543 (470–625)	500 (392–595)	NS
Median PW percentile	28.9 (18.3–57.2)	12.6 (2.9–35.0)	.0095
Fetal-to-placental ratio			
Median (IQR)	4.48 (3.79–4.91)	6.05 (5.38–6.47)	<.0001
>90th percentile	1 (3.6)	7 (29.2)	.018
<10th percentile	9 (32.1)	1 (4.2)	.014

BW percentiles calculated according Fetal Medicine Foundation, and PW percentiles and fetal-to-placental ratios calculated according to Thompson et al.^14^ Data are presented as number (percentage) or median (IQR), unless otherwise indicated. In comparison between the group with gastroschisis and the group with omphalocele, the Fisher exact test was used for categorical variables, and the Mann-Whitney test was used for continuous variables. A *P* value of <.05 is considered significant.

*BW*, birthweight; *IQR*, interquartile range; *NS*, not significant; *PW*, placental weight; *SGA*, small for gestational age.

Amylidi-Mohr. Discordant birthweight: placental weight ratios in fetal gastroschisis and omphalocele. Am J Obstet Gynecol Glob Rep 2023.

**Figure 2:**
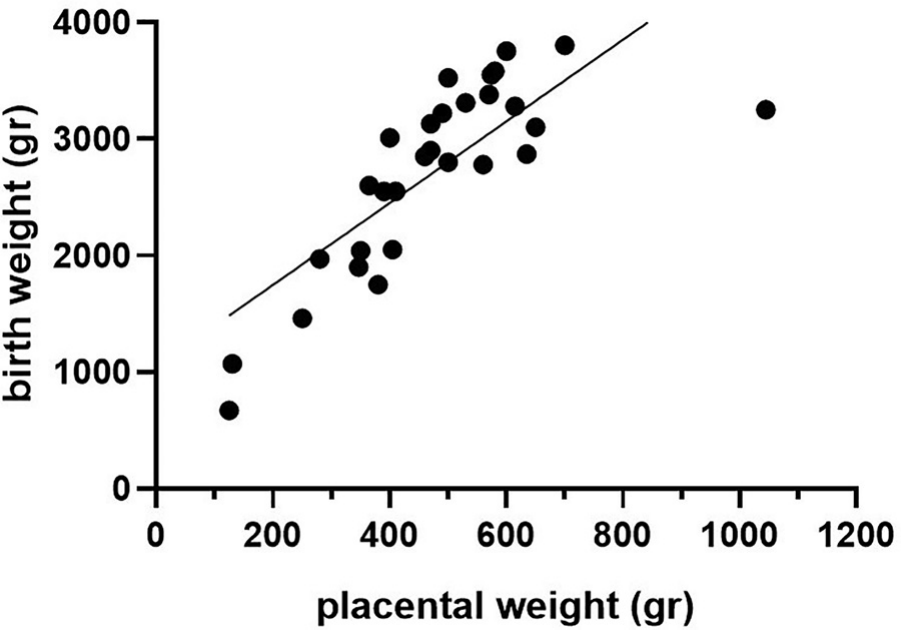
Correlation of birthweight and placental weight in children born with omphalocele

Of the 24 newborns diagnosed with omphalocele, BWs and PWs are shown in Table 2. Of 24 children, 4 (16.7%) were born SGA (<10th percentile), and all children had a small placenta <10th percentile. There is a significant correlation of BW to PW (*P*<.0001) (Figure 2).

Amylidi-Mohr. Discordant birthweight: placental weight ratios in fetal gastroschisis and omphalocele. Am J Obstet Gynecol Glob Rep 2023.

**Figure 3:**
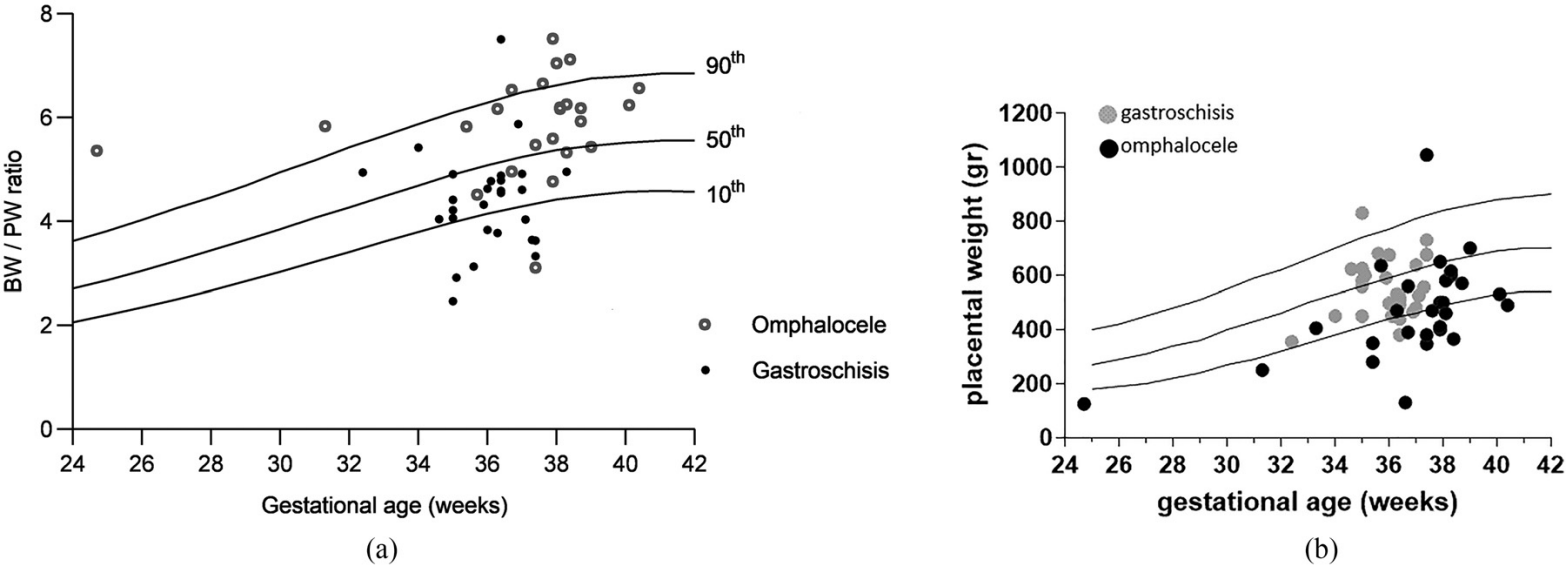
Placenta weight and BW/PW ratio according to gestational age A, Distribution of BW-to-PW ratios among children born with gastroschisis and omphalocele. PWs plotted on reference ranges derived from Thompson et al.^14^ The *lines* represent the 10th, 50th, and 90th percentiles for gestational age. B, Observed PW–to–expected PW ratio according to gestational age. *BW*, birthweight; *PW*, placental weight.

**Figure 4:**
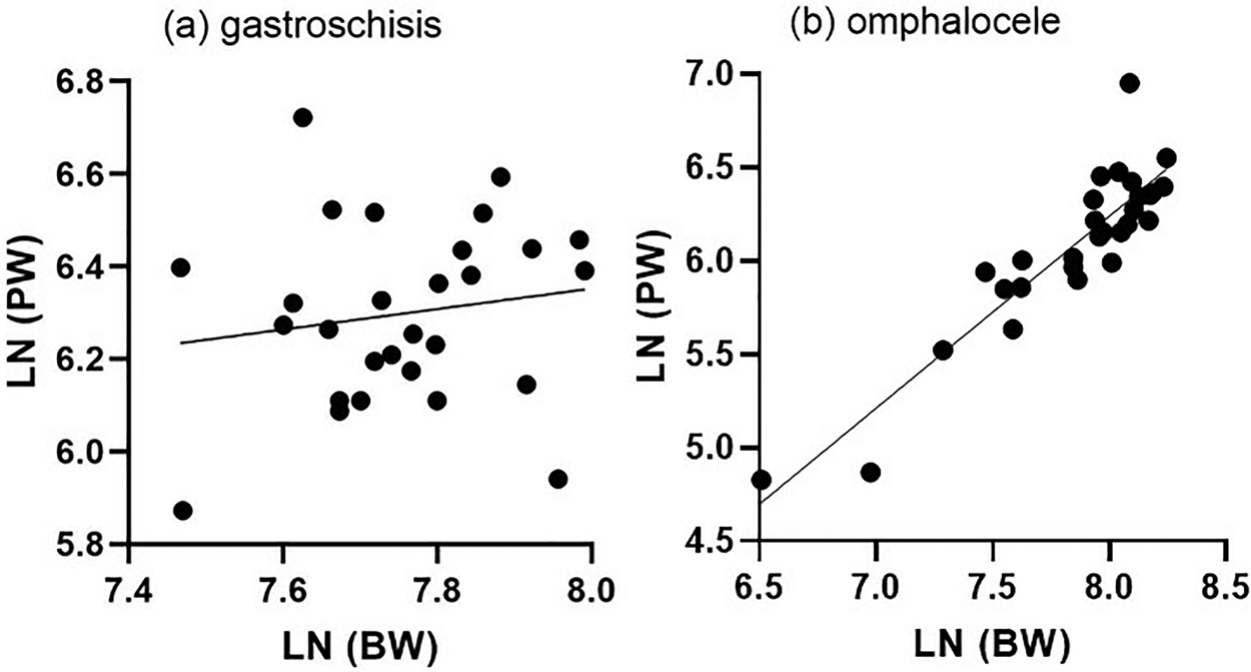
Relationship between birthweight and placental mass *Fitted straight lines* represent the LNs of BW and PW in (**A**) fetuses with gastroschisis and (**B**) fetuses with omphalocele. *BW*, birthweight; *LN*, natural logarithm; *PW*, placental weight.

The BW-to-PW ratio differs significantly between pregnancies diagnosed with gastroschisis and pregnancies diagnosed with omphalocele (4.48 [3.79–4.91] vs 6.05 [5.38–6.47], respectively; *P*<.0001) (Figure 3). The median BW percentile in children with gastroschisis is significantly lower than in children with omphalocele (*P*=.0013). Conversely, the PW percentile is higher in pregnancies with gastroschisis than in pregnancies with omphalocele (28.9 [18.3–57.2] vs 12.6 [2.9–35.0], respectively; *P*=.0095). Linear regression modeling of the population with omphalocele revealed a metabolic scaling exponent β of 0.8399 to 1.216 (R^2^=0.82) and Ln (PW)=−3.461 to −0.5064 × Ln (BW). Linear regression modeling of the population with gastroschisis revealed a metabolic scaling exponent β of −0.3440 to 0.7865 (R^2^=0.02) and Ln (PW) = −0.1972 to 8.967 × Ln (BW) (Figure 4).

Amylidi-Mohr. Discordant birthweight: placental weight ratios in fetal gastroschisis and omphalocele. Am J Obstet Gynecol Glob Rep 2023.

Amylidi-Mohr. Discordant birthweight: placental weight ratios in fetal gastroschisis and omphalocele. Am J Obstet Gynecol Glob Rep 2023.

## Discussion

### Principal findings

All diagnosed cases of AWDs in our department over a 19-year period between 2001 and 2021 were retrieved. After excluding growth-related factors, such as chromosomal aberrations and other malformations, we focused on the growth development of both fetuses with gastroschisis and omphalocele and their placentae. This study aimed to investigate the source of growth restriction and determine whether the principle of allometric metabolic scaling applies in those 2 groups. This study demonstrates that children with gastroschisis are often born small for GA, although their placentas are normal in weight and no correlation exists between BW and PW. The BW-to-PW ratio in children born with gastroschisis is significantly lower than in children born with omphalocele, who are often born with a normal BW but have smaller placentas and therefore a high BW-to-PW ratio. In children with omphalocele, PW correlates to BW.

### Results

The BW-to-PW ratio has been mooted as a predictor of long-term health,^19^ and where morphologic and functional placental adaptation occurs, these adaptations will affect the composition of the developing fetus.^20^ The concept of BW-to-PW ratio as a marker of placental nutrient transfer efficiency is also strongly supported by studies in mice, with clear evidence of placentas adapting their nutrient transfer capacity according to their size. In humans, the data are less conclusive in a normal population concerning the system.^5^ A Norwegian study demonstrated that both low and high BW-to-PW ratios have an increased risk of fetal death in preterm deliveries, whereas, at term, an elevated risk of fetal demise was found in the highest quartile.^21^ This finding suggests that a small placenta concerning the neonatal size could be a risk factor for fetal death at term. Regarding metabolic scaling, we found a lower scaling exponent β value in gastroschisis and a higher scaling exponent β value in omphalocele compared with normal pregnancies. A higher value of β correlates with a newborn weight lower than that predicted by Kleiber's metabolic scaling law. It is assumed that a deviation from a β of 0.75 may reflect decreased metabolic efficiency of the placenta, as β reflects the fractal structure of the placental vasculature.^7^^,^^17^ This correlates with the elevated BW-to-PW ratio in omphalocele but does not explain the cases in gastroschisis.

### Clinical implications

In growth restriction because of uteroplacental insufficiency, we would expect a consistent correlation between neonatal weight and PW. In children without genetic or structural defects, the correlation of placental parameters, such as weight, thickness, and surface to fetal weight, has been studied quite extensively, and low PW was found to be associated with FGR.^22^, ^23^, ^24^

In fetuses with omphalocele, the significant correlation of PW to fetal weight supports the hypothesis that, similar to healthy fetuses, smallness is an expression of placental insufficiency. Why many children with omphalocele are normal size although their placentas are small remains unclear. In children with congenital heart defects, a possible explanation for placental dysfunction is that early changes in the fetal circulation influence placental development.^25^ Therefore, a possible explanation for the low PWs might be the altered fetoplacental circulation in children with omphalocele because of the herniated visceral organs compromising umbilical blood flow. Another explanation might be a relative fetal overgrowth because of altered expression of genes involved in cell growth, as often found in children with Beckwith-Wiedemann syndrome, a distinct genetic anomaly also associated with omphalocele. However, to date, we are not aware of any findings supporting either explanation.

However, in children with gastroschisis, no such correlation is found, and other mechanisms seem to impair fetal growth. In addition, Stoll et al^26^ reported that placental size was not reduced in a smaller series of gastroschisis cases. A possible explanation could be the protein loss across the extruded bowel loops, as described by Carroll et al,^27^ who found elevated amniotic fluid protein concentration and reduced cord blood protein concentrations in gastroschisis. An etiology other than uteroplacental insufficiency may explain that the rate of IUD of 4% is lower than previously described, and the prevalence does not seem to increase in late pregnancy.^28^^,^^29^ In addition, there is evidence that gestational hypertension is less common in the mothers of patients with gastroschisis, another finding that gastroschisis is not associated with placental malfunction, as the placenta plays a central role in the development of gestational hypertension.^30^ However, other studies suggest that placental histology and dysfunction might contribute to growth restriction. Comparing patients with gastroschisis and controls, chorangiosis and severe villous edema were more common in patients with gastroschisis.^31^ Chorangiosis represents fetal hypoxemia and the placenta's attempt to improve gas exchange across the terminal villi and takes weeks to develop.^32^

Nevertheless, a study has shown that the outcome of infants with gastroschisis and intrauterine growth restriction (IUGR) is no different from that of infants without IUGR when they are stable in utero (ie, neither dilation of the middle cerebral artery nor constant reversal of umbilical artery flow during diastole). Furthermore, infants born prematurely with gastroschisis have increased gastrointestinal morbidity compared with those born at term. Moreover, in the proper environment and without the other major morbidities, infants with IUGR behave similarly to other infants with gastroschisis.^11^ However, whether elective preterm delivery improves the overall outcome of children born with gastroschisis remains contradictory.^9^^,^^10^

### Research implications

It seems important to continue research on the placental defensive and potentially altered adaptive mechanisms in fetuses with AWD. Analyzing PW, BW-to-PW ratio, and the scaling exponent ß may offer additional clues to understanding the processes at the maternal-placental interface.

### Strengths and limitations

The strength of our study is that all cases were diagnosed, managed, and delivered in 1 referral tertiary center. Despite the retrospective data, the analysis included cases with a full dataset gathered prospectively. Moreover, this study investigated an altered metabolic scaling value in pregnancies complicated by AWD. A limitation of this study is the high number of LTFU. However, many of the cases of LTFU were diagnosed with chromosomal anomalies and would not qualify for this study.

## Conclusions

Our data raise concern that pregnancies with suspected growth restriction and gastroschisis may more likely undergo indicated preterm delivery, which might result in higher prematurity-related morbidity. Although more data are needed, there is evidence to suggest that intervention is justified only if FGR is accompanied by fetal compromise, in particular in a preterm pregnancy.
